# Revision of unicondylar knee arthroplasty: an analysis of failure rates and contributing factors

**DOI:** 10.1186/s43019-025-00276-3

**Published:** 2025-05-23

**Authors:** Dominik Szymski, Josina Straub, Nike Walter, Yinan Wu, Oliver Melsheimer, Alexander Grimberg, Volker Alt, Arnd Steinbrueck, Markus Rupp

**Affiliations:** 1 Department of Trauma Surgery, University Medical Centre Regensburg, Regensburg, Germany; 2 Deutsches Endoprothesenregister gGmbH (EPRD), Berlin, Germany; 3 Orthopädisch Chirurgisches Kompetenzzentrum Augsburg (OCKA), Augsburg, Germany; 4 Department of Trauma, Hand and Reconstructive Surgery, University Hospital Giessen, Klinikstrasse 23, 35385 Giessen, Germany

**Keywords:** Knee, Revision, Infection, Arthroplasty, Unicompartimental, Loosening

## Abstract

**Background:**

The implantation rate of knee arthroplasty and, in particular of unicondylar knee arthroplasty (UKA), is increasing, and revision is a feared complication. The aim of this study was to identify factors influencing aseptic and septic revision that are of high interest for establishing preventive measures.

**Methods:**

Data were collected using the German Arthroplasty Registry (EPRD). Patients with UKA were analyzed using the multiple Log-rank test with Holm’s method. Septic and aseptic revisions were calculated using Kaplan–Meier estimates. In total, 300,998 cases of knee arthroplasty were identified in the registry, and 36,861 patients with UKA were analyzed with a maximum follow-up of 7 years.

**Results:**

The primary reason for UKA revision surgery was aseptic loosening (32.5%), particularly loosening of the tibial component (19.0%), followed by infection (11.0%) and the progression of arthritis (10.0%). Over 7 years, 8.7% of UKA procedures required revision, 7.8% for aseptic causes and 0.9% for infection. Risk factors for aseptic revision included uncemented implants [hazard ratio (HR) 1.38] and low annual surgical volume (fewer than 25 UKAs/year, HR 1.86; fewer than 50 UKAs/year, HR 1.43). Significant risks for septic revision were grade III obesity (HR 1.83), male sex (HR 1.69), and high comorbidity scores (Elixhauser > 5, HR 1.67). The surgical volume did not affect septic revision rates.

**Conclusion:**

Aseptic loosening is the primary cause of UKA revision, influenced by implant type and low surgical volume, while septic revisions are associated with patient factors such as obesity, male sex, and comorbidities. Improvements in implant selection, surgical expertise, and patient risk management may reduce revision rates.

**Level of evidence:**

III, retrospective case–control study.

## Introduction

The changing demographic landscape with an increasingly aging population is leading to a rising incidence of osteoarthritis of the knee in society. Consequently, there has been a significant increase in knee arthroplasty procedures [18], encompassing both total knee arthroplasty (TKA) and unicondylar knee arthroplasty (UKA). TKA is a long-established procedure, and the use of UKA has also shown a continuous upward trend. Despite the growing popularity and the well-documented success of UKA, its long-term outcomes and failure rates continue to be debated. Literature reports have described up to three times higher revision rates for UKA compared to TKA. Murray and Parkinson recommend that knee surgeons use at least 20% UKA in their arthroplasty procedures to minimize the risk of revision through appropriate technique and diagnosis [14].

Understanding the multifaceted factors that contribute to UKA failure is essential to formulating effective strategies to improve implant longevity and optimize patient outcomes. As the prevalence of UKA continues to rise, it is imperative to comprehensively assess the reasons for revision surgery, whether aseptic or septic, to better inform health care providers and improve implant longevity. By identifying the specific factors associated with UKA failure, clinicians and researchers can develop targeted strategies to minimize risk, improve implant longevity, and ultimately improve patient outcome. Compared with findings from international registries such as the UK National Joint Registry (NJR) and the Australian Orthopaedic Association National Joint Replacement Registry (AOANJRR), the present data from the German EPRD highlights both shared and unique risk factors for UKA revision, particularly with respect to fixation method preferences and surgical volume distribution.

The aim of this study was therefore to (1) analyze the rate of aseptic and septic revision of UKA for osteoarthritis of the knee and to (2) identify the factors influencing revision surgery.

## Material and methods

### Data collection

This study is based on the prospective “German Arthroplasty Registry” (Endoprothesenregister Deutschland; EPRD) and examines all types of revision surgery of UKA in patients with primary gonarthrosis. All hip and knee replacements performed in Germany since 2012 have been documented in the “German Arthroplasty Registry” (EPRD) in collaboration with the statutory health insurance funds (AOK Bundesverband GbR, Verband der Ersatzkassen e.V vdek), the German Medical Technology Association (BVMed), and several participating hospitals. To date, more than 2 million procedures have been included in the registry, and approximately 70% of all hip and knee arthroplasty procedures performed in Germany were included in the registry by 2022 [8]. The data provided by surgeons are cross-validated by including two participating health insurance companies (AOK-B, vdek), covering approximately 65% of the German population. Surgical revisions registered in the EPRD are tracked using insurance billing data, even if they are performed at a hospital not participating in the arthroplasty registry. With the exception of procedures performed outside of Germany, this algorithm ensures near-perfect tracking of patients insured by these companies [9].

The German versions of the International Classification of Procedures in Medicine (ICPM), the “Operation and Procedure Code” (OPS) 301 system, and the 10th Revision of the International Classification of Diseases (ICD-10) were used to classify and identify diagnoses and procedures. The study was approved by the Ethics Committee of the University of Kiel (ID: D473/11) and was conducted in accordance with the Declaration of Helsinki. Informed consent was obtained from all patients. Biological sex was categorized as male or female based on hospital records. All data were fully anonymized in accordance with German data protection law.

### Patients

All patients over 18 years of age with primary osteoarthritis of the knee as main diagnosis (ICD-10: M17.0-, M17.1) who underwent UKA between November 2012 and September 2022 were included in the present analysis of the German Arthroplasty Registry (EPRD). Patient characteristics such as age, sex, body-mass-index (BMI), Elixhauser comorbidity score, and American Society of Anesthesiologists (ASA) score are recorded in the registry, as well as hospital-related factors such as the surgical volume of the implantation. The Elixhauser score is an index that pools a variety of comorbidities of different organ systems and entities [24]. Coded comorbidities during the initial hospitalization for primary arthroplasty were the basis for calculating the Elixhauser score. The BMI was divided into underweight (< 20 kg/m^2^), normal weight (20–25 kg/m^2^), pre-obese (25–30 kg/m^2^), obesity grade I (30–35 kg/m^2^), obesity grade II (35–40 kg/m^2^, and obesity grade III (> 40 kg/m^2^). The reason for revision was determined by searching the ICD-10 code for aseptic cause (T84.4) or periprosthetic infection (T84.5) in the registry and by surgeon input. According to the guidelines of the European Bone and Joint Infection Society (EBJIS), a definition of PJI was obtained from the surgeons and coded as PJI and therefore recorded as septic failure in the registry [11]. The surgeons detailed the reason for aseptic revision in a standardized form via the registry. Analysis of the “Operation and Procedure Code” (OPS codes) provided a detailed record of the procedure. The data provided by the surgeons were cross-validated by analysis of the insurance data. Hospital size was provided by the GBA (Gemeinsamer Bundesausschuss). Exclusion criteria were patients who were not treated for primary osteoarthritis as the main diagnosis. Patients with a follow-up of less than 12 months or with an implant other than UKA were also excluded from data collection. Patients without clear information on the material used were also excluded from the analysis. A minimum follow-up period of 12 months was chosen to ensure adequate data completeness while capturing early revision events, particularly those related to fixation failure, and to reduce potential bias from early postoperative censoring.

### Statistical analysis

The data were analyzed to identify aseptic and septic revision rates and their influencing factors in UKA in Germany. The statistical program R (R Foundation for Statistical Computing version 4.2, Vienna, Austria) was used for statistical analysis. Categorical variables were presented as frequencies and percentages. Comparison between septic and aseptic revision was carried out using the corrected Multiple Log-rank test with Holm’s method to adjust for multiple comparisons.

Subsequently, cumulative incidences for revision surgery were calculated using Kaplan–Meier estimates. Categorical variables are presented as number of observations and frequency, while continuous variables are presented as mean and standard deviation. The significance level was assessed at the 5% level.

## Results

This study included 36,861 patients with UKA for primary osteoarthritis of the knee from the German Arthroplasty Register (EPRD). Anthropometric patient specific characteristics and hospital-related properties are summarized in Table 1.

**Table 1:** Patient and hospital characteristics

Characteristic	UKA, *N* = 36,861
BMI	
Underweight	41 (0.2%)
Normal	3469 (14.0%)
Pre-obese	9325 (38.0%)
Obese I	7280 (30.0%)
Obese II	3231 (13.0%)
Obese III	1309 (5.3%)
Unknown	12,206
Age, years	
< 55	5,943 (16%)
55–64	13,373 (36%)
65–74	10,645 (29%)
75 +	6,900 (19%)
Sex	
Female	20,796 (56%)
Male	16,065 (44%)
ASA	
1	1235 (15%)
2	5184 (63%)
3 +	1830 (22%)
Unknown	28,612
Elixhauser score	
< 0	8460 (23%)
0	20,607 (56%)
1–4	3308 (9.0%)
5 +	4486 (12%)
UKA implant volume per year	
0–25	9175 (25%)
26–50	7761 (21%)
51–200	9811 (27%)
200 +	9535 (26%)
Unknown	579

*UKA* Unicondylar arthroplasty, *ASA* American Society of Anesthesiologists Score

### Cause of failure

The most common reason for revision surgery was aseptic loosening (total 32.5%), in particular loosening of the tibial part (19.0%). Infection (11.0%) and arthritis progression (10.0%) were other frequently reported reasons for UKA revision (Table 2).

**Table 2:** Revision causes for unicondylar arthroplasties

Revision causes	*N* = 2411^1^
Aseptic loosening (femur)	62 (3.5%)
Aseptic loosening (tibia)	324 (19.0%)
Aseptic loosening (patella)	3 (0.2%)
Aseptic Loosening (multiple)	171 (9.8%)
Periprosthetic joint infection (PJI)	197 (11.0%)
Progression of arthritis	175 (10.0%)
Ligament instability	98 (5.6%)
Periprosthetic fracture	90 (5.1%)
Movement restriction	85 (4.9%)
Failure of an implant component	81 (4.6%)
Osteolysis with fixed implant (femur)	4 (0.2%)
Osteolysis with fixed implant (tibia)	3 (0.2%)
Osteolysis with fixed implant (multiple)	2 (0.1%)
Condition after prosthesis removal	49 (2.8%)
Misalignment/rotation error	47 (2.7%)
Implant wear	36 (2.1%)
Other reasons	323 (18.0%)
Unknown	661

^1^*n* (%)

### Revision rates

**Fig. 1:**
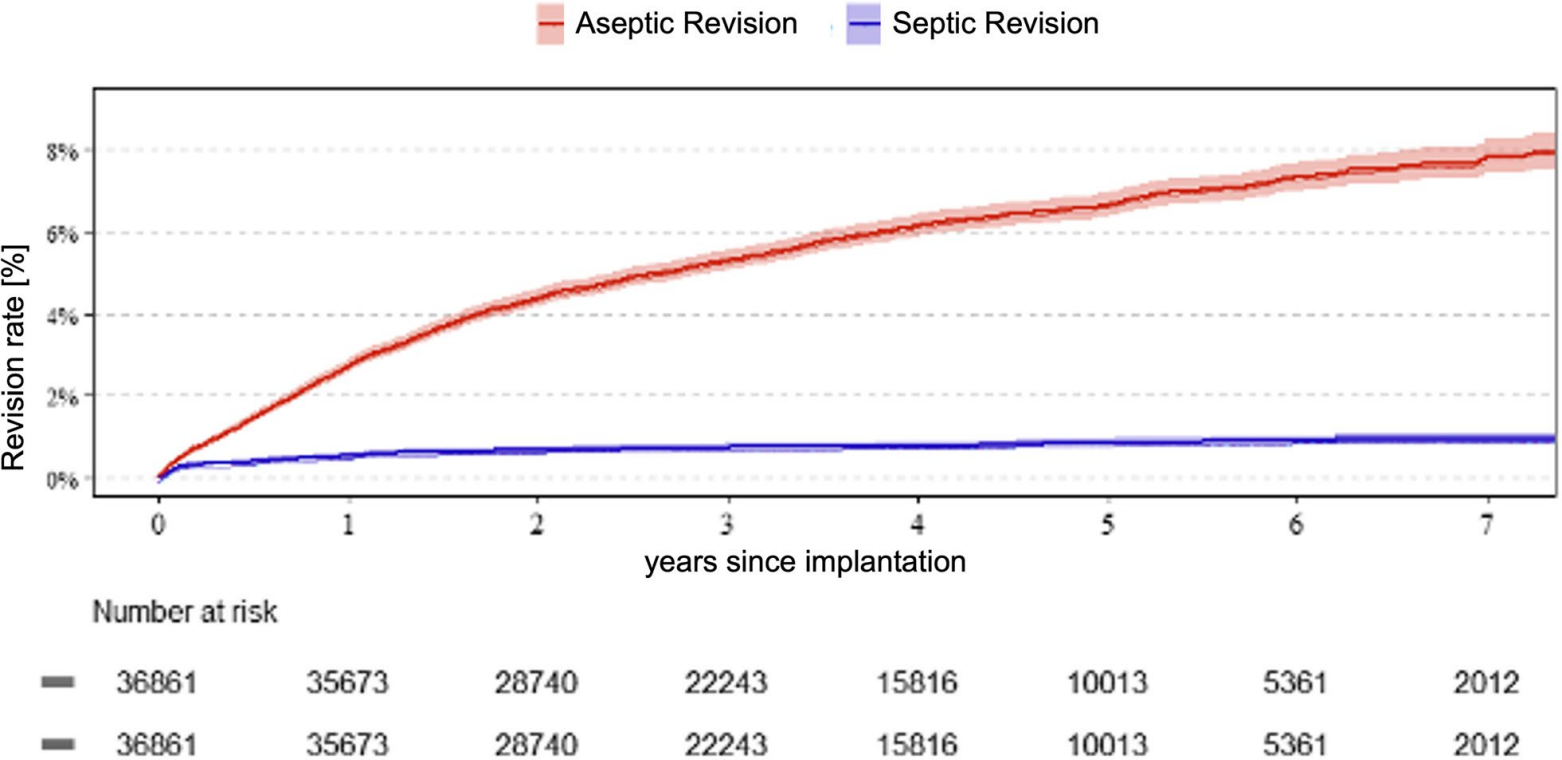
Rate of failure for aseptic and septic reasons of unicondylar knee arthroplasty in the German Arthroplasty Register (EPRD)

After 7 years, 8.7% of all UKAs required either septic or aseptic revision. After 1 year, 2.7% of all UKAs required aseptic revision; this rate was 6.6% after 5 years and 7.8% after 7 years. Owing to periprosthetic joint infection (PJI), 0.5% of UKAs were revised after 1 year, 0.9% after 5 years, and 0.9% after 7 years (Fig. 1).

### Risk factors

Influencing factors for aseptic revision were uncemented arthroplasty [hazard ratio (HR) 1.38; 95% confidence interval (CI) 1.15–1.66, *p* < 0.001], an implant volume of less than 25 UKAs per year (HR: 1.86, 95% CI 1.58–2.19, *p* < 0.001), and an overall implant volume of less than 50 (HR: 1.43, 95% CI 1.22–1.69, *p* < 0.001). Influencing factors for septic revision were obesity grade III (HR: 1.83, 95% CI 1.04–3.24, *p* = 0.037), male sex (HR: 1.69, 95% CI 1.26–2.26, *p* < 0.001), and an Elixhauser score > 5 (HR: 1.67, 95% CI 1.06–2.65, *p* = 0.029). Septic revision was not affected by implant volume (Tables 3, 4).

**Table 3:** Influencing factors for aseptic revision of UKA

Characteristic	HR^1^	95% CI^1^	*p*-Value
Age, years			
< 55	–	–	
55–64	0.77	0.66–0.90	< 0.001
65–74	0.75	0.64–0.88	< 0.001
75 +	0.70	0.57–0.85	< 0.001
BMI			
Obese I	–	–	
Underweight	1.24	0.40–3.86	0.7
Normal	0.96	0.80–1.16	0.7
Pre-obese	0.86	0.75–1.00	0.047
Obese II	0.95	0.79–1.13	0.5
Obese III	1.13	0.89–1.42	0.3
Fixation			
Cemented	–	–	
Hybrid	0.95	0.52–1.72	0.9
Uncemented	1.38	1.15–1.66	< 0.001
Sex			
Female	–	–	
Male	0.75	0.66–0.84	< 0.001
UKA implant volume			
51–200	–	–	
0–25	1.86	1.58–2.19	< 0.001
26–50	1.43	1.22–1.69	< 0.001
200 +	0.85	0.71–1.03	0.10
Elixhauser score			
< 0	–	–	
0	0.87	0.75–1.00	0.057
1–4	0.83	0.66–1.04	0.10
5 +	0.87	0.71–1.08	0.2
Hospital size			
Small (< 250 beds)	–	–	
Medium (251–500 beds)	1.20	0.99–1.46	0.062
Large (> 500 beds)	1.47	1.20–1.81	< 0.001

^*1*^*HR* hazard ratio, *CI* confidence interval

**Table 4:** Influencing factors for septic revision of UKA

Characteristic	HR^1^	95% CI^1^	*p*-Value
Age, years			
< 55	–	–	
55–64	1.11	0.72–1.70	0.6
65–74	1.23	0.78–1.94	0.4
75 +	0.94	0.54–1.61	0.8
BMI			
Obese I	–	–	
Underweight	3.80	0.52–27.6	0.2
Normal	0.96	0.58–1.60	0.9
Pre-obese	0.94	0.64–1.37	0.8
Obese II	1.47	0.95–2.26	0.082
Obese III	1.83	1.04–3.24	0.037
Fixation method			
Cemented	–	–	
Hybrid	1.20	0.30–4.88	0.8
Uncemented	0.83	0.47–1.45	0.5
Sex			
Female	–	–	
Male	1.69	1.26–2.26	< 0.001
UKA implant volume			
51–200	–	–	
0–25	1.39	0.93–2.08	0.10
26–50	1.03	0.69–1.55	0.9
200 +	0.60	0.36–1.02	0.058
Elixhauser score			
< 0	–	–	
0	0.79	0.53–1.17	0.2
1–4	1.21	0.72–2.04	0.5
5 +	1.67	1.06–2.65	0.029
Hospital size			
Small (< 250 beds)	–	–	
Medium (251–500 beds)	1.15	0.72–1.83	0.5
Large (> 500 beds)	1.05	0.64–1.75	0.8

^*1*^*HR* hazard ratio, *CI* confidence interval

## Discussion

In this analysis of revision surgery on the basis of the German Arthroplasty Registry (EPRD), the evaluation of more than 36,000 unicondylar knee arthroplasties showed a clear predominance of aseptic reasons for failure. Both patient-specific and clinic-specific influencing factors, such as obesity, an increased number of comorbidities, and a low implant volume in the treating hospital were relevant for both septic and aseptic replacements.

The main reason for revision surgery within the first seven years after UKA implantation was aseptic, particularly aseptic loosing, which accounted for nearly one-third of all revisions. Loosening of the tibia was reported in 19.0% of all revisions in the EPRD. Other major aseptic reasons of UKA revision surgery were progression of arthritis (10.0%) and ligament instability (5.6%). Revisions due to septic failure were responsible for 11.0% of revisions. Previous studies on unicondylar arthroplasty also reported aseptic failure as the main reason for revision surgery [6]. Similar to our data, Mikkelsen et al. reported in the Danish Endoprosthesis Registry revision rates of up to 10.0% within 10 years for aseptic reasons and a significantly increased revision risk compared to total arthroplasty [12]. Similar to the EPRD data, an analysis of US data from 2001 to 2010 showed instability (32.2%) and aseptic loosening (22.9%) as the main reasons for aseptic revision [16]. Burger et al. identified osteoarthritis progression (22.3%) as the most common reason for aseptic revision of cemented UKA in the Dutch registry between 2007 and 2018, followed by loosening of the tibial component (20.3%) and malalignment (15.3%) [6]. Similar results were reported by Ekhtiari et al. in a cohort study conducted in Canada that identified a 10-year revision prevalence of 16.5%, which corresponds to our data. Similarly, the most common reason for revision surgery was mechanical loosening in nearly 84.0% of all reported UKA revisions [7]. In our data, septic revisions were significantly less frequent and also significantly less likely than total knee arthroplasty (TKA), which has also been described in the literature [3, 22, 23].

Relevant risk factors for aseptic UKA revision were uncemented fixation of the prosthesis, low UKA implant volume of the treating hospital, and a larger hospital size as identified in the analysis of the cases in the German Arthroplasty Registry. This result supports the recommendation by Murray and Parkinson that UKA be used in at least 20% of all arthroplasties to minimize the risk of revision through appropriate technique and correct diagnosis and indication [14]. The observed association between low surgical volume and increased revision risk underscores the importance of centralizing UKA procedures in high-volume centers, enhancing surgical training, and implementing quality assurance measures to improve patient outcomes. Previous literature reports also described several other risk factors. Similar to our results, Ekhtiari et al. found an increased risk of revision for uncemented arthroplasty, diabetes mellitus, male sex, and age younger than 50 years [7]. Several other reports confirmed these reported risk factors for aseptic revision in patients with UKA [2, 10, 16, 17].

For septic revision, obesity, male sex, and a high comorbidity rate were reported as relevant risk factors for the development of PJI in patients with UKA, which have already been described in previous literature. Significant influencing factors for the development of infection described by Blanco et al. were obesity with a BMI greater than 30 (odds ratio (OR): 8.86), diabetes mellitus (OR: 2.33), and comorbidities, which were measured as ASA score of III or IV (OR: 15.3) [5]. Septic revision remains a significant burden for both patients and the healthcare system [19, 25]. While our findings are grounded in the German healthcare system, the identified risk factors and revision patterns may be generalizable to other systems with comparable registry structures and surgical practices, though differences in implant selection and patient populations should be considered when interpreting these results.

Preoperative preparation should include the evaluation of the Elixhauser score, BMI, and comorbidities to aid in patient selection and optimization. High-risk patients, such as patients with severe obesity or significant comorbidities, should be closely monitored pre- and postoperatively. The use of dual antibiotic-loaded bone cement may also be an option to minimize the rate of PJI [4, 20]. Surgeons at low-volume centers should engage in additional training and adhere to standardized protocols to improve outcomes. Encouraging case-sharing arrangements, in which complex cases are referred to centers with high levels of expertise, may further mitigate the volume-outcome disparity [14]. Prehabilitation and weight management programs may also improve outcome in obese patients, while systemic diseases such as diabetes mellitus should be tightly controlled preoperatively [1, 13]. Whenever possible, cemented techniques should be the preferred method, particularly in patients with high-risk profiles such as younger age or high activity levels. The use of cement has consistently demonstrated superior results in reducing early aseptic loosening [10, 15, 17].

Despite the advantages of using the German Arthroplasty Registry, there are certain limitations owing to the study design. Differences in the indications for the implants investigated resulted in different quantities reported. Corrected multiple log-rank test and Kaplan–Meier estimates were used to address this issue. Data quality depends on the accuracy of registration by surgeons and the correct coding during registration. The registry includes cross-validated insurance data to mitigate this limitation. However, the history of the registry currently prohibits follow-up beyond seven years, despite evidence that most septic failures occur within this timeframe [21]. The calculation of the Elixhauser score used comorbidities coded at the time of initial hospitalization, which are potential confounders if coded incorrectly or inadequately. While volume per hospital has been identified as a risk factor, the EPRD does not report volume per surgeon. Further studies are necessary to analyze the effect of surgeon volume compared with hospital volume.

## Conclusion

Aseptic revision surgery, such as aseptic loosening and progression of osteoarthritis, represents the main reason for documented revisions in the German Arthroplasty Register (EPRD) for UKA. Analysis of the EPRD identified patient-specific factors, such as obesity and comorbidities, and clinic-specific factors, such as a low implantation rate, as risk factors for aseptic revision. Relevant risk factors should be identified preoperatively, potentially modifiable factors should be improved, and patients at risk should be closely monitored.

## Data Availability

Data available on request.
